# African and classical swine fever: similarities, differences and epidemiological consequences

**DOI:** 10.1186/s13567-017-0490-x

**Published:** 2017-11-28

**Authors:** Katja Schulz, Christoph Staubach, Sandra Blome

**Affiliations:** 1 Friedrich-Loeffler-Institut, Federal Research Institute for Animal Health, Institute of Epidemiology, Südufer 10, 17493 Greifswald, Insel Riems Germany; 2 Friedrich-Loeffler-Institut, Federal Research Institute for Animal Health, Institute of Diagnostic Virology, Südufer 10, 17493 Greifswald, Insel Riems Germany

## Abstract

For the global pig industry, classical (CSF) and African swine fever (ASF) outbreaks are a constantly feared threat. Except for Sardinia, ASF was eradicated in Europe in the late 1990s, which led to a research focus on CSF because this disease continued to be present. However, ASF remerged in eastern Europe in 2007 and the interest in the disease, its control and epidemiology increased tremendously. The similar names and the same susceptible species suggest a similarity of the two viral diseases, a related biological behaviour and, correspondingly, similar epidemiological features. However, there are several essential differences between both diseases, which need to be considered for the design of control or preventive measures. In the present review, we aimed to collate differences and similarities of the two diseases that impact epidemiology and thus the necessary control actions. Our objective was to discuss critically, if and to which extent the current knowledge can be transferred from one disease to the other and where new findings should lead to a critical review of measures relating to the prevention, control and surveillance of ASF and CSF. Another intention was to identify research gaps, which need to be closed to increase the chances of a successful eradication of ASF and therefore for a decrease of the economic threat for pig holdings and the international trade.

## Introduction

Similar names suggest similar disease characteristics for African and classical swine fever (ASF and CSF). In fact, ASF was even thought to be caused by the same virus as CSF [1] before Montgomery [2] described it as an independent disease entity in Kenya. Yet, despite the similar clinical signs and some shared pathogenic characteristics, the two diseases are caused by completely different viruses [1, 3, 4].

Nonetheless, both diseases are frequently mentioned at the same time or compared to each other, especially when it comes to epidemiology and disease control. They are both listed by the World Organization for Animal Health (OIE). Diseases found on this list are of considerable international interest and subject to specific regulations [5]. ASF as well as CSF are viral diseases affecting pigs (*Suidae*) exclusively. In the case of an outbreak, both diseases may generally entail substantial economic consequences for the affected country or region, particularly in western European countries with a considerable pig industry [6–10].

Up to very recently, most central and eastern European countries had mainly experience with CSF, and in many cases, control strategies for ASF were copied from CSF-contingency plans of the past. However, the recent developments of the ASF epidemics in the Baltic EU Member States and in Poland showed that the disease dynamics did not follow the expected pattern and several open questions remain [11, 12]. The disease neither died out nor spreads with high speed as predicted [13]. So far, the affected countries encounter new cases every week and the situation is out of control in the wild boar population. In this review, our focus was put on similarities and differences of the two viral diseases and the subsequent epidemiological consequences. Due to the particular difficulties to control the diseases in the wild boar population and the constant threat, the presence of the virus in wild boar poses to domestic pig holdings, we focused on the epidemiology in wild boar. By including the latest available scientific findings, this review may help to improve our understanding of the epidemiology of CSF and ASF and thus to optimize prevention and control measures. Furthermore, existing uncertainties were identified and thereby new research can be inspired.

## Virus

### ASF

#### Virus taxonomy and morphology

The ASF virus (ASFV) is a large enveloped double-stranded deoxyribonucleic acid (DNA) virus and the only DNA arbovirus (arthropod borne) known so far. The virus belongs to the *Asfarviridae* family; genus *Asfivirus* [14]. The genome consists of a linear double-stranded DNA molecule of 170–190 kbp with terminal inverted repetitions and hairpin loops [15]. The viral genome codes for more than 50 structural proteins and several non-structural proteins. ASFV molecular polymorphism has been investigated by partial sequencing of the gene encoding the major capsid protein p72, and 22 distinct genotypes were defined [16]. Recently, an additional genotype was described by Gallardo et al. [17]. Additional sequence information is gathered through partial sequencing of the B602L gene (CVR) or the gene encoding p54. The virus strains involved in the current eastern European outbreaks belong to genotype II and are highly identical. They show so far only very minor differences. The virus strains circulating on Sardinia are of genotype I and also showed only minor variability, even after decades. In the study of Frączyk et al. [18], they identified genetic variability within genes related to evasion of host immune system. According to Frączyk et al. [18] this could help tracing the direction of ASFV isolates molecular evolution. However, studies, identifying further new genetic markers are clearly needed that allow higher resolution molecular epidemiology and thus outbreak tracing.

#### Clinical signs and pathology

The occurrence and the manifestation of clinical signs depend on different factors. Decisive factors can for example be the virulence of the virus strain, the infection route and dose and the constitution of the affected animal. The incubation period is described to be 2–7 days [19]. According to Sanchez-Vizcaino et al. [20] it can be 5–15 days. Peracute, acute, subacute and chronic form of disease can be distinguished [20]. The ASFV strains causing the outbreaks in eastern Europe are highly virulent and the clinical courses are usually acute and lethal [17, 21]. Experimentally infected wild boar showed also a very high mortality, independently of sex or age [21, 22]. This does not preclude very unspecific courses that can almost go unnoticed. Some characteristics of the different disease forms are outlined in Table 1.

**Table 1:** **Characteristics of the four manifestations of an infection with the African swine fever virus**

	Peracute form	Acute form	Subacute form	Chronic form
Virulence	High	High/moderate	Moderate	Low
Clinical signs	High fever, appetite loss, lethargy, hyperpnoe	High fever, appetite loss, lethargy, gastro-intestinal signs	See acute form but less pronounced	Respiratory signs, lameness
Pathology	Erythema	Erythema, petechial haemorrhages in several organs, lung oedema, abortion	Erythema, petechial haemorrhages in several organs, haemorrhagic lymph nodes, abortion	Arthritis, necrotic skin, pneumonia, pericarditis, abortion
Mortality	High	High	Variable	Low

Partly adapted from Sanchez-Vizcaino et al. [20]

As described in Table 1, mortality may vary according to the virulence of the ASF virus. Infections with high virulent virus strains usually lead to 90–100% mortality.

#### Immune response and vaccination

Pigs recovering from ASFV infection are usually protected against homologues challenge, but cross-protection against heterologous strains is often missing. Generally, the existence of an antibody-mediated protection, i.e. virus neutralization, is controversially discussed. It is possible to confer a certain level of protection by passive transfer of hyperimmune sera [23]. However, several authors suggest the complete absence of neutralizing antibodies [24], others found that antibodies could reduce virus titers or neutralize ASF virus to a certain extent in vitro [25–27].

It has been reported that animals surviving ASF can become long-term carriers [28, 29]. This may have a tremendous impact in wild boar populations. So far, it is not clear how many of the survivors may act as carriers and how long they remain infectious. Evidence exists indicating that at least not all animals become long-term carriers [30].

While the role of antibodies is controversially discussed, cytotoxic T-cell responses seem to play a major role in mediating antiviral protection. It was demonstrated that depletion of CD8+ cells leads to abrogation of protection [31].

Safe and efficacious vaccines against ASF do so far not exist, although several approaches have been pursued to develop immunization protocols [32]. Thus, a control strategy in both domestic pigs and wild boar has to rely on veterinary hygiene.

### CSF

#### Virus taxonomy and morphology

The agent causing CSF is a small, positive single-stranded, enveloped RNA virus. The CSF virus (CSFV) belongs to the genus *Pestivirus* within the Flaviviridae family [33]. The genome consists of approximately 12.3 kb and includes one large open reading frame (ORF) flanked by two non-translated regions (NTRs) [34–36]. The viral genome codes for eleven viral proteins, four structural and seven non-structural (NS) proteins. In detail, the core (C) protein along with three envelope glycoproteins (E1, E2, and Erns) constitutes the virion, and Npro, p7, NS2-3, NS4A, NS4B, NS5A, and NS5B are NS proteins [37, 38].

CSFV strains can be assigned to three distinct genotypes with three to four subtypes [39–41]. This classification is based on the nucleotide sequences of fragments of the 5′-non-translated region (5′-NTR), and of the region encoding the glycoprotein E2 [39, 42]. Different subtypes show a particular geographical distribution and genetic typing is used to understand both gross and molecular epidemiology [39, 43, 44]. Recent European strains belong to genotype 2, especially subtypes 2.1 and 2.3. Most often, these virus strains are moderately virulent.

#### Clinical signs and pathology

Also for CSF, the course of disease depends on several factors like viral virulence, virus dose, health status and particularly the age of the affected animal. Three different courses of infection are known, namely the acute, chronic and prenatal form. The latter can lead to the so called “late onset” form [7, 45]. The incubation period is in the range of 4–10 days. The acute form of CSF manifests often in fever, respiratory and gastro-intestinal signs, lethargy, and inappetence. The acute lethal form can be accompanied by severe hemorrhagic or neurological signs. Mortality in piglets can be very high, whereas older animals can withstand an infection and develop a life-long immunity [46].

The chronic form is caused by viruses with a lower virulence and usually effects unspecific symptoms like runting, secondary infections of both respiratory and gastro-intestinal tract, skin lesions, and, in the case of sows, reduced fertility. Sometimes, animals can show an initial recovery, however after several months all animals succumb to infection and die. During the whole time of infection, the affected animals shed large amounts of virus [46, 47]. This course can play an important role in the maintenance of virus transmission.

The outcome of transplacental infection depends on the stage of gestation. In early pregnancy, CSFV infection usually causes abortion, still birth, mummification or malformation [47]. However, infections in the 2nd and 3rd month of pregnancy may lead to the development of persistently infected piglets. These piglets are immunotolerant towards the causative virus strain and may be born healthy. However, they usually runt and develop the so-called late onset form of CSF. Also, these animals constantly shed virus until they eventually die [45, 47, 48].

Regarding the pathology of acute forms, lymph nodes, spleen and kidneys as well as other organs may be edematous and hemorrhagic. Moreover, spleen infarctions and necrotic regions in the tonsils are sometimes found. In animals dying due to the chronic form of CSF, the typical hemorrhages are usually missing, while necrotic lesions in the gastrointestinal tract are more common [47]. Secondary infections may dominate the pathological lesions. The same is true for the late-onset form [49].

#### Immune response and vaccination

Protection against CSFV upon vaccination or an overcome infection is mediated by both humoral and cellular immune responses. Animals that have recovered from field virus infection and animals vaccinated with a conventional live-attenuated vaccine develop antibodies against the structural proteins E2 and Erns as well as the non-structural protein NS3 [50–52]. Especially the E2 antibodies are able to neutralize CSFV and antibody titers can be determined using cell culture-based neutralization assays [53]. Measurable titers are usually found between days 14 and 21 post infection and persist probably lifelong. Moreover, antibodies are transferred by immune sows to their offspring via colostrum. These antibodies have a half-life of roughly 12–14 days and are able to passively protect suckling piglets for a couple of weeks [54]. Beside humoral responses, cell-mediated immunity plays an important role in early protection upon vaccination and in beneficial immune responses upon field virus infection.

Safe and efficacious vaccines exist for both intramuscular vaccination of domestic pigs and oral vaccination of wild boar [55]. The latter have proven that they can be an important tool for CSF eradication from affected wild boar populations [56].

## Epidemiology

### ASF

#### Transmission and contagiosity

Three main transmission cycles are described for ASF [57]. A distinction is made between the sylvatic cycle, the tick-pig cycle, and the domestic cycle. The sylvatic cycle refers to the circulation between the African wild suid population and soft ticks. This cycle can be seen in African countries where ASF and ticks of the genus *Ornithodoros* are endemic. The tick-pig cycle is present in Africa and played a role on the Iberian Peninsula, where ticks infested pig pens and shelters. In the domestic cycle, direct or indirect transmission occurs between domestic pigs. The same applies to transmission among wild boar in the sylvatic cycle in eastern Europe [57, 58]. Direct contact between infected and susceptible animals is a very effective transmission route, but still depending on the virulence of the virus [28, 59]. Indirect transmission is described through people, vehicles etc. [60]. Although officially banned in most European countries, feeding contaminated meat products or fodder to wild boar or domestic pigs is assumed to play a considerable role in the transmission of ASF [61]. The introduction of the ASF virus from Africa to Portugal in 1957 as well as the introduction into Georgia in 2007 happened most likely through swill feeding of waste from ships at international harbors [62]. ASF virus could be found in boar semen, therefore a transmission through sexual contact or artificial insemination cannot be ruled out [63]. According to Penrith and Vosloo [64] there is no evidence for intrauterine transmission. This is in line with our own unpublished observations.

Ferreira et al. [65] detected viral DNA in air samples and showed a significant association between the detection of virus in feces and in air samples. However, due to the high virus load needed, airborne transmission is not thought to be a major transmission route for ASFV.

Infected animals excrete virus through body fluids like blood, nasal fluid and through feces and urine. However, the amount of virus differs in different fluids. Several studies demonstrated a considerable virus burden in the blood of infected animals, while it was considerably lower in nasal or rectal fluids [22, 58, 66]. Accordingly, contact to infectious blood appears to be the most effective transmission route for ASF [19]. Also, Depner et al. [13] hypothesized that due to the necessary direct contact, the contagiosity of ASF is lower than previously assumed. Results of experimental and field studies support this hypothesis [22, 67]. Following infection studies, the oral infectious dose can vary between 10 000 and 18 000 TCID_50_ (50% tissue culture infective dose) [68].

Virus transmission can be described by the basic reproductive number (R_0_), which defines the number of secondary infected animals that result from one infected animal. Existing data about the R_0_ value for different ASF virus strains varies considerably in different studies, ranking from 0.5 to 18.0. However, independently of the virus strain, R_0_ was generally lower when transmission happened only through indirect contact [22, 58, 59].

#### Vectors and carriers

In addition to domestic pigs, wild suids play an important role in the transmission pathways of ASF. In Africa, especially warthogs and bush pigs are known as an asymptomatic reservoir for ASFV [60]. Transmission between warthogs has not been described so far: the presence of soft ticks is therefore believed to be necessary for the spread of the disease [69]. The epidemiological role of other African wild suids such as giant forest hogs in the distribution of ASF has not been conclusively evaluated [57]. Many studies demonstrated that the European wild boar is as susceptible to ASF as domestic pigs and can thus act as reservoir under European conditions [69].

As described further above, ASFV is an arbovirus that can replicate in soft ticks. In areas, where ticks of the *Ornithodoros* genus are endemic, they can play an important role in the transmission of the ASFV [57, 58]. There is no indication that birds or rodents from infected farms contracted ASF [58]. These findings could be confirmed by Penrith and Vosloo [64]. Mellor et al. [70] could experimentally transmit ASFV from Stomoxys flies to pigs. For central Europe, there is no evidence that soft ticks could play a role [71]. There is no evidence that *Ornithodoros* spp. occur in this region. Moreover, hard ticks do not seem to play a role either [72].

#### Tenacity

It is known that the survival time of the virus can be up to 18 months in serum at room temperature. However, the survival time decreases with increasing temperature and can be longer in frozen material. The virus is stable across a wide range of pH-levels; it can resist a pH level between 4 and 13 [73]. Several studies demonstrated that ASFV can stay infective in raw ham or sausage but also in treated meat products for several months. However, it was also shown, that cooking meat kills the virus within few minutes, whereas it can stay infectious at least 1000 days in frozen meat [74–76].

### CSF

#### Transmission and contagiosity

Virus can be excreted through feces and all body fluids like saliva and urine. Infected animals may excrete large amounts of virus over a relatively long period [77]. Infection usually happens oro-nasally often through direct but potentially also through indirect contact [7, 78, 79]. The infectious dose through oro-nasal infection ranges between 10 TCID_50_ and 80 TCID_50_ [65]. Different indirect transmission routes are described. Indirect contact to wild boar, for example through contact to contaminated hunting material or vehicles could be identified as an important source for virus introduction into commercial pig holdings [80, 81]. Also, indirect transmission through infected feed or garbage (illegal swill feeding) has been suggested as a common source for virus introduction into a naïve population [7, 80]. Movements of persons entail the risk of transmission through contaminated clothes, vehicles or repeatedly used needles [82–84]. Indirect transmission via excretions are described to be rather unlikely [85].

The CSFV is able to cross the placental barrier and consequently to infect fetuses in the uterus [45, 86]. Virus transmission through boar semen has also been reported [87–89]. Transmission via air was suspected in farms where secondary outbreaks without any detectable direct or indirect contact to the originally affected farm have occurred [82]. Potential virus transmission via air could be documented under experimental conditions [15, 90, 91]. Weesendorp et al. [92] and Weesendorp et al. [93] detected CSF virus in a pen where infected pigs had been housed. However, Weesendorp et al. [91] showed that the transmission rate was significantly higher among pigs housed in the same pen then between pigs housed in different pens or via air, which emphasizes the importance of the transmission routes mentioned above.

The R_0_ value for CSF virus depends on the number of susceptible animals, on the population density and also on the virulence of the CSF virus [7, 45, 94]. Several studies determined a high R_0_ values for within-herd transmission, indicating a high contagiosity when direct contact between the animals is possible [86, 95, 96]. However, Weesendorp et al. [94] showed that direct transmission is highly dependent on the virulence of the virus strain. They found that pigs that had direct contact with animals infected with a low virulent strain did not get infected.

Besides the direct relationship between population density and the R_0_, a reduced number of highly susceptible young pigs decreases the chance of disease persistence in a population [7, 97–99]. Stegeman et al. [100] found that the transmission of CSF virus among breeding pigs was clearly lower with a R_0_ of 2.9 than in herds of weaned piglets and slaughter pigs.

#### Vectors and carriers

Although the role of various animal species as potential vectors for CSF has been intensively studied, transmission seems to occur mainly if not exclusively between pigs. Neither arthropods nor rodents or birds could be reliably identified as vectors for the virus [82, 101, 102]. Wild boar constitute an important carrier of CSFV and therefore pose a constant risk to introduce the virus into pig farms [7, 80]. Everett et al. [103] showed in their study that warthogs as well as bushpigs can be infected with CSF virus and can also transmit the disease.

#### Tenacity

Similar to ASFV, the tenacity of CSFV in the environment depends on a number of factors. Several studies could demonstrate a relationship between ambient temperature and the tenacity of the virus [77, 104–107]. Accordingly, the period of time, the virus remains infectious, decreases with increasing temperature. In the study of Weesendorp et al. [104] it was calculated that virus would remain infectious for a few days in feces and urine at 22 °C. However, at 5 °C infectious virus would remain detectable for several weeks. Botner and Belsham [108] could show that the tenacity of CSF virus in slurry was short when it was heated, but the virus remained infectious for weeks at cool temperatures.

Farez and Morley [107] describe in their study a tenacity of years in meat frozen at −70 °C. They also listed time periods, for which the virus stayed infectious in different meat products, illustrating that these periods may range from 40 days to several years, depending on the treatment. Treatments like salt-cures and smoking do not seem to reduce the infectivity of CSF virus significantly, whereas pasteurization and cooking inactivates the virus [105]. Also, the protein concentration in the matrix influences the tenacity of the virus. The higher the protein concentration, the longer stays the CSF virus infectious [77]. Another factor affecting the stability of the virus is the pH-value [105–107, 109]. It was found that virus is inactivated below a pH-level of 4 and above pH 11 [109].

## History and today’s distribution

### ASF

**Figure 1:**
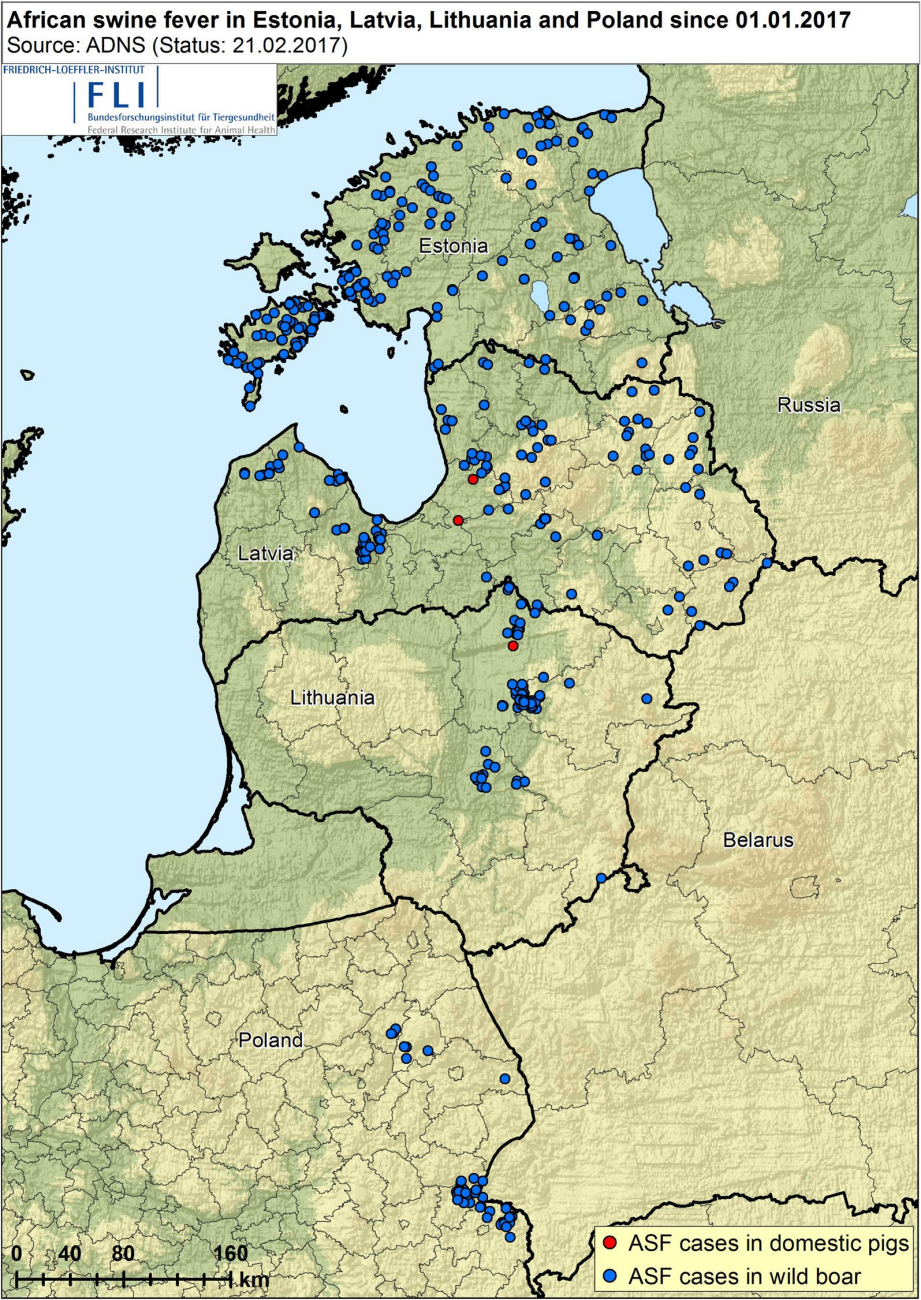
**Current distribution of African swine fever cases in domestic pigs and wild boar in the affected countries of the European Union**. (Source: ADNS Data, Status 21.2.2017).

The first time, when ASF was identified as an independent disease entity, was in Kenya in 1910 [2]. After its first detection, ASF was found to circulate in several African states until it was introduced into Portugal in 1957. After successful eradication in Portugal, the disease was reintroduced in 1960 and spread to several European countries. Before it was finally eradicated in 1995, ASF stayed endemic on the Iberian Peninsula [6, 61, 64]. Since the virus was newly introduced into Sardinia in 1978, ASF has remained endemic in several parts of Sardinia [110]. The disease did not only reach Europe, but also different countries in South and Central America, from where it was successfully eradicated. For many years, ASF could be found endemic only in African states and Sardinia [61]. However, in 2007 ASF was again detected in Europe, namely in Georgia, from where it spread into the neighbor states Armenia, Azerbaijan and the Russian Federation [62, 111]. In 2012 and 2013, also the Ukraine and Belarus reported an ASF outbreak [20]. In 2014, ASF reached the European Union, where outbreaks were confirmed in Lithuania, Latvia, Estonia and Poland [11, 12, 20, 112]. Currently, the virus is still circulating in all four countries with frequent new outbreaks, mainly in wild boar, but occasionally also in domestic pigs (Figure 1). In addition, ASF cases were detected in Moldova for the first time in October 2016 [113].

### CSF

The first official reports about the occurrence of CSF virus originate from Ohio, USA, where the disease was first described in 1833. Between 1860 and 1970 the CSF was widely distributed over the American and the European continents [105]. In 1978, CSF was eradicated in North America [109]. Since then, North America and Australia are officially free from CSF [114]. Mainly due to inadequate reporting and lack of surveillance, the disease situation in Africa remains unclear. However, it is known, that CSF has been endemic in parts of Asia as well as in areas of Central and South America since several years [114]. After devastating outbreaks in the Netherlands and in Germany in the late 1990s and sporadic outbreaks that occurred thereafter, the last outbreaks in Europe were reported in domestic pigs from Latvia in 2014. In wild boar, however, the disease was at least present until 2016 in the latter country [115].

## Prevention and control measures

### ASF

Currently, no vaccination for ASF is available. To prevent the introduction of ASF, movement restrictions regarding pigs, pork, blood and other products from pigs kept in affected areas as well as potentially contaminated material, vehicles etc. are in place. Following European Commission [116], necessary biosecurity measures are defined, e.g. swill feeding, in commercial pig farms as well as in wild boars, must be prohibited, especially in high risk areas. Direct or indirect contact to wild boar or to any by products has to be avoided. The measures that have to be taken in a case of ASF suspicion or an actual outbreak in the European Union have been specified by European Commission [117]. When an outbreak of ASF in a farm has been confirmed, all pigs of the premise must be culled. In addition, further measures like the safe disposal of all potentially contaminated material, restriction (minimum radius of 3 km) and surveillance (minimum radius of 10 km) zones with movement restrictions for pigs and products of porcine origin have to be set up. Specific regulations have been defined for both zones in European Commission [117].

### CSF

The prevention and control measures regarding CSF in domestic pigs are very similar to the ones described for ASF. Detailed regulations applying for member states of European Commission [118]. However, in the case of specific epidemiological situations, vaccination can be used to control CSF in domestic pigs. Vaccination of wild boar can be also be applied and may represent the method of choice in combination with other elements of surveillance and control [118].

## Conclusions

Following the introduction of ASF into the Trans-Caucasian countries and the Russian Federation in 2007 and into the European Union in 2014, several countries including Germany sought to set up and update their surveillance and control plans (contingency plans) for the disease.

Especially the countries with previous CSF experience tried to use their CSF contingency plans as a blue print and copied most of the measures that had been found suitable to control CSF.

For the control measures of ASF in wild boar populations, this approach does not seem to be promising as the disease dynamics proved to be too different for the two diseases: Neither self-limitation, which was assumed to occur due to the high virulence of the virus strain circulating in Eastern Europe nor fast spread due to high contagiosity and connected habitats took place [13]. Thus, reconsideration of control and surveillance options is needed.

In this review, we tried to point out major similarities and differences of CSF and ASF with the overall objective to provide background information on disease biology and dynamics that could feed into adapted strategies. Some of the most important similarities/differences are summarized in Table 2.

**Table 2:** **Summary of the most important differences and similarities between African swine fever (ASF) and classical swine fever (CSF)**

	ASF	CSF	Both diseases
Virus			
Virus taxonomy and morphology	Large DNA virus	Small RNA virus	
Clinical signs and pathology			Among others high fever, appetite loss, lethargy, erythema, petechiae
Immune response and vaccination	Lack of neutralizing antibodies, no or insufficient cross-protection among strains, protection linked to cytotoxic T-cell responses No vaccination available	Existence of neutralizing antibodies, cross-protection among genotypes, safe and efficacious vaccines available	
Epidemiology			
Transmission and contagiosity			Direct and indirect transmission
	Most effective with blood contact, no evidence for intrauterine transmission	Virus shedding with all se- and excretions, intrauterine transmission and resulting persistent infection of fetuses possible	
Vectors and carriers			Wild boar important reservoir
	Transmission through ticks possible	No transmission through arthropods or rodents described	
Tenacity			Long infectivity in cold environmental temperatures
History and today’s distribution	For long time only endemic in Africa and Sardinia since 2007 present in Europe	Long-term epidemics in wild boar over the last decades, sporadic occurrence in domestic pigs; currently no outbreaks in domestic pigs, no cases reported in wild boar	
Prevention and control measures	No vaccination	Effective vaccination	
			High biosecurity, no swill feeding, no contact between domestic pigs and wild boar

The similarities mainly concern the range of vertebrate hosts as well as clinical signs and pathomorphological lesions that necessitate swift and reliable diagnostic tools. Both diseases are usually accompanied by a steep increase in mortality when introduced into a naïve population. This gives passive surveillance high impact for the early detection of disease introduction into both domestic pigs and wild boar [119]. With regard to the detection and differentiation of the diseases, molecular tools have been developed and validated that allow both steps in one assay (e.g. [120, 121]). Moreover, both routine sample sets and alternative sample matrices work for both diseases with quite similar performance [122].

Another similarity is the quite high tenacity of the causative agents, especially under cold conditions [74, 76, 105]. Both viruses, ASFV and CSFV, are able to remain infectious for several weeks under adequate climatic conditions (cold environment). Elevated temperatures inactivate both viruses rather quickly. Moreover, both are stable within a wide range of pH-values [73, 109].

Apart from these basic features, which could at least lead to combined passive surveillance approaches in disease free areas that are at risk, several differences exist between ASF and CSF that take effect especially when wild boar populations are concerned.

### Epidemiologically relevant facts concerning CSF

Recent European CSFV strains have shown moderate virulence and an age-dependence of clinical symptoms. This is important for the target population of active surveillance but also disease dynamics as it can be assumed that older animals will survive [119]. Survivors will be safe as they are protected probably livelong from reinfection. Immune sows will confer protection to young piglets via maternally derived antibodies in the colostrum.

In outbreak regions with moderate to high wild boar density, the seroprevalence often rises very quickly and antibody detection is a most valuable tool to characterize the outbreak extent.

Long-term shedders will most probably be present (chronically infected animals and persistently infected piglets after transplacental transmission), but meet increasing population immunity. Shedding is generally high in all se- and excretions and thus, swift spread is likely within a sounder.

Also, CSF has shown potential to become endemic in wild boar populations rather than dying out. This is probably due to the high wild boar density in affected areas in Europe in combination with the above mentioned low/moderate virulence. For this virus, this virulence level could be an optimum for long-term maintenance [123]. However, vaccination exists as an additional tool to eradicate CSF from a wild boar population and most probable, even production and application of a DIVA (differentiation of infected from vaccinated animals) vaccine is feasible [58].

### Epidemiologically relevant facts concerning ASF

Recent European ASFV strains have shown high virulence [124], almost no age-dependence of clinical symptoms and a high case-fatality ratio [19]. The fate of survivors is still not clear as these animals could act as long-term carriers. In fact, survivors will at least be positive for prolonged periods [28, 125]. In the later stages of their infection, mobility can be assumed and thus possible increase in infectious contacts. However, there is also evidence that this is not inevitable [30].

In outbreak regions, the seroprevalence is rising steadily but slowly. It often stays below 10%, even in heavily affected areas. Thus, serology is an important tool to understand and investigate disease dynamics but a difficult target for active surveillance (sample sizes that could detect seropositivity with a sufficiently low prevalence threshold and acceptable confidence can hardly be obtained).

Shedding is generally low in most se- and excretions and thus, blood contact is the main source of infection. Even within groups of animals that have close contact, transmission might be slow and some animals may even go uninfected within a highly affected sounder. Yet, due to the high tenacity of the virus in blood, infectiousness can be assumed for long periods and thus, carcasses and blood contaminated fomites can act as long-term source of infection. Transplacental transmission has not been described for ASF [64].

Little is known about the role of maggots or other insect larvae, the fate of carcasses under different conditions, and environmental factors such as so soil underneath a carcass. It could recently be demonstrated that several of these matrices are positive for ASFV genome, but live virus is probably rare or non-existent.

Although not involved in the current situation in Eastern Europe, soft ticks can play a role in ASF transmission. This may add another player and more complexity to the control scenario. It has been proven that tick involvement can have high impact on outbreak duration.

No vaccine exists that could aid control options. Developing a vaccine for the wild boar population would mean to develop a safe and efficacious oral vaccine. So far, there is no such vaccine at the horizon.

Thus, besides the shared common features, the differences between ASF and CSF clearly dominate and entail more serious epidemiological consequences. With regard to surveillance actions, the focus for CSF on piglets is clearly counterproductive for the current ASF situation. For ASF, herd immunity does not play an important role for a long period of time and thus time does not act necessarily as beneficial factor. CSF and ASF have different levels of contagiosity and thus transmission characteristics, for example, the R_0_ for ASF is lower than for CSF. However, there is a relatively low number of studies, in which these values were estimated. Moreover, different algorithms, virus strains, diagnostic tools and host characteristics were used, which makes those studies hardly comparable. Nonetheless, experimental as well as field studies refute previous assumptions of a high contagiosity of ASF. Based on the low contagiosity but high tenacity of the virus in carcasses and blood, ASF surveillance has to focus even more on detecting dead individuals to avoid any direct contact and therefore further spread [126].

Regarding ASF, further studies should focus on ASF transmission in the field and on environmental factors, like soil and organisms around wild boar carcasses. Moreover, the role of survivors needs further investigation.

One of the research gaps concerning CSF relates to the final licensing of the available DIVA vaccine. The use of such a vaccine would help to better understand the balance between vaccine induced and natural immunity and thus dynamics of epidemics and their control.

To close these gaps and to deduce appropriate control options, collaboration is needed among research institution of affected and non-affected countries.

## Abbreviations

ASF: African swine fever
CSF: classical swine fever
DNA: deoxyribonucleic acid
RNA: ribonucleic acid
TCID_50_: 50% tissue culture infective dose
R_0_: basic reproductive number
DIVA: differentiating infected from vaccinated animals
